# Hydrophobicity Improvement of Cement-Based Materials Incorporated with Ionic Paraffin Emulsions (IPEs)

**DOI:** 10.3390/ma13143230

**Published:** 2020-07-20

**Authors:** Jinyang Huo, Zhenjun Wang, Haoyan Guo, Yongfeng Wei

**Affiliations:** 1 Research and Development Center of Transport Industry of Technologies, Materials and Equipments of Highway Construction and Maintenance. (Gansu Road & Bridge Construction Group), Lanzhou 730030, China; 2019031005@chd.edu.cn (J.H.); wei_yong_feng@sina.com (Y.W.); 2 School of Materials Science and Engineering, Chang’an University, Xi’an 710061, China; hyguo@chd.edu.cn

**Keywords:** ionic paraffin emulsions (IPEs), hydrophobicity improvement, impermeability, apparent contact angle, correlation analyses

## Abstract

Cement-based materials are non-uniform porous materials that are easily permeated by harmful substances, thereby deteriorating their structural durability. In this work, three ionic paraffin emulsions (IPEs) (i.e., anionic paraffin emulsion (APE), cationic paraffin emulsion (CPE), and non-ionic paraffin emulsion (NPE), respectively) were prepared. The effects of incorporation of IPEs into cement-based materials on hydrophobicity improvement were investigated by environmental scanning electron microscopy (ESEM), Fourier transform infrared (FTIR) spectroscopy, transmission and reflection polarizing microscope (TRPM) tests and correlation analyses, as well as by compressive strength, impermeability, and apparent contact angle tests. Finally, the optimal type and the recommended dose of IPEs were suggested. Results reveal that the impermeability pressure and apparent contact angle value of cement-based materials incorporated with IPEs are significantly higher than those of the control group. Thus, the hydrophobicity of cement-based materials is significantly improved. However, IPEs adversely affect the compressive strength of cement-based materials. The apparent contact angle mainly affects impermeability. These three IPEs impart hydrophobicity to cement-based materials. In addition, the optimal NPE dose can significantly improve the hydrophobicity of cement-based materials.

## 1. Introduction

Cement-based materials are non-uniform porous materials that are easily permeated by harmful substances in water, thereby deteriorating their structural durability. Hydrophobicity is a key index to evaluate impermeability, corrosion resistance and durability of cement-based materials [1,2,3,4,5,6]. Hydrophobicity improvement of cement-based materials is effective to improve their corrosion resistance and durability via the decrease of their permeability, which deserves extensive attention [7,8,9,10,11].

The hydrophobicity of cement-based materials is predominantly improved by surface hydrophobic modification and internal hydrophobic modification [12,13,14,15,16,17,18,19]. Generally, surface hydrophobic modification involves the surface treatment of cement-based materials, such as coating, dipping and spraying. Previously, a hydrophobic release agent with SiO_2_-CH_3_ submicron-sized particles has been developed [12]; this agent increases the apparent contact angle of the cement mortar surface to higher than 145°, which can improve the hydrophobicity of cement mortar without reducing its compressive strength. Super-hydrophobic concrete has been developed by spraying ethylene–vinyl acetate copolymer to improve its structural property [16]. A silica-based hybrid nanocomposite, SiO_2_/polymethylhydrosiloxane (SiO_2_/PMHS), has been used for the surface treatment of cement-based materials, which can reduce their water absorption and air permeability [17]. However, surface hydrophobic modification of cement-based materials exhibits limitations. Once the surface hydrophobic layer of cement-based materials is destroyed, the newly exposed part becomes hydrophilic, leading to the rapid decrease in their hydrophobicity.

On the other hand, internal hydrophobic modification involves the addition of hydrophobic substances during the mixing process of cement-based materials. As-prepared cement-based materials exhibit excellent hydrophobicity. Even if the surface of cement-based materials is destroyed, the newly exposed part still exhibits hydrophobicity. Common hydrophobic additives include silane, siloxane, stearic acid, penetron admix, and waste rubber powder [1,18,19]. A low-cost waterborne stearic acid emulsion has been introduced to improve the hydrophobicity and corrosion resistance of concrete [18]. However, the mechanical strength of concrete reportedly decreases. In addition, octyltriethoxysilane-based materials have been used for improving the hydrophobicity and durability of concrete [19]. However, the high cost of octyltriethoxysilane-based materials hinders their application. Therefore, it is imperative to prepare new cost-effective, suitable, and high-strength hydrophobic additives.

In this work, self-prepared ionic paraffin emulsions (IPEs) with excellent dispersion and high stability were used to improve the hydrophobicity of cement-based materials. The hydrophobicity of cement-based materials was improved by the combination of surface hydrophobic modification and internal hydrophobic modification. The compressive strength, impermeability, apparent contact angle, and microstructures of specimens were investigated and compared with those of the control group. The effect of IPEs on the hydrophobicity improvement of cement-based materials was analyzed. Meanwhile, correlation analyses among compressive strength, apparent contact angle, and impermeability were established. In addition, the optimal type and the recommended dose of IPEs were investigated to improve the hydrophobicity of cement-based materials. The objective of this work was examination of the hydrophobicity improvement of cement-based materials incorporated with IPEs. This work is of scientific interest for concrete compositions used in areas where corrosion acts with water as a support.

## 2. Experimental

### 2.1. Materials

Composite Portland cement, fine aggregate, fully refined paraffin, ionic emulsifiers (anionic, cationic, and non-ionic types), carboxymethylcellulose calcium, carrageenan, and mixing water (tap water, deionized water) were used in this work. Tap water was used to prepare the specimens, and deionized water was used to prepare self-prepared IPEs. Table 1, Table 2 and Table 3 show the properties of composite Portland cement, fine aggregate, and fully refined paraffin, respectively.

### 2.2. Preparation of Self-Prepared Ionic Paraffin Emulsions (IPEs)

**Figure 1:**
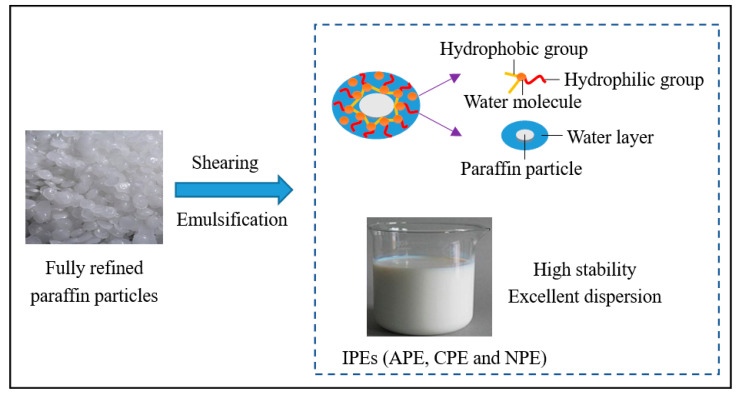
Preparation of self-prepared ionic paraffin emulsions (IPEs).

Figure 1 shows the preparation of self-prepared IPEs. First, 36% fully refined paraffin particles, 5% ionic emulsifiers (anionic type: alkyl alcohol polyether sulfate; cationic type: cetylpyridinium bromide; non-ionic type: alkylphenol polyoxyethylene ether formaldehyde condensate), 0.5% carboxymethylcellulose calcium, 0.5% carrageenan, and 58% deionized water were placed in beakers; and then these beakers were placed in a bath with 60 °C water. A beaker containing paraffin particles was removed from the water bath when paraffin particles were molten and the temperature was constant. Then, the deionized water heated to 60 °C was poured into a colloid mill for preheating. Second, molten paraffin particles, ionic emulsifier, carboxymethylcellulose calcium, and carrageenan were gradually poured into the colloid mill. Third, the glass rod was used for one-way stirring to aid in the shearing and emulsification of the colloidal mill. Finally, emulsions were cooled to 20 °C, affording self-prepared IPEs.

### 2.3. Preparation of Specimens

Two specimens were used in this work: cement paste and cement mortar specimens. Among these specimens, cement paste specimens were used for the compressive strength and environmental scanning electron microscopy (ESEM) tests, while cement mortar specimens were used for the impermeability test. Table 4 and Table 5 show the mix proportions of cement paste specimens and cement mortar specimens, respectively.

First, for cement paste specimens, IPEs and water were mixed at a slow speed (140 r/min) for 10 s using a mixer, followed by the slow addition of cement. The mixture was mixed at a slow speed (140 r/min) for 120 s and then at a high speed (285 r/min) for 120 s. For cement mortar specimens, IPEs and water were mixed at a slow speed (140 r/min) for 10 s using a mixer, followed by the slow addition of cement. The mixture was mixed at a slow speed (140 r/min) for 60 s, followed by the even addition of the fine aggregate (the feeding time of the fine aggregate was not longer than 30 s). The mixture was mixed at a slow speed (140 r/min) for 120 s and then at a high speed (285 r/min) for 120 s. Second, for cement paste specimens, the mixture was poured into molds (40 mm × 40 mm × 160 mm) and cured at 20 °C. For cement mortar specimens, the mixture was poured into frustum-shaped molds (top diameter of 70 mm, bottom diameter of 80 mm, and height of 30 mm) and cured at 20 °C. After 24 h, two specimens were demolded and placed into a standard curing room for 28 days. Third, surfaces of two specimens were polished using sandpaper and cleaned with a wet cloth after drying for 48 h in an oven at 60 °C. Then, IPEs were coated on surfaces of these two specimens by a coating machine. The thickness of the dry film coating as measured by a high-magnification digital microscope was about 350 µm. Finally, two specimens were dried indoors for 7 days.

### 2.4. Test Methods

#### 2.4.1. Fourier Transform Infrared (FTIR) Spectroscopy Test

Compositions of IPEs were analyzed by Fourier transform infrared (FTIR) spectroscopy (Bruker; Karlsruhe, Germany). First, 5 mL of IPEs was placed in beakers. Then, beakers were placed in an oven at 50 °C for 2 h to remove the water in IPEs. Second, beakers were removed until IPEs were cooled to 20 °C, followed by grinding IPEs into particles. Finally, IPE particles were placed on the sample table. FTIR diffuse reflection spectra were recorded from 4250 cm^−1^ to 750 cm^−1^ to examine the functional groups of the IPE particles.

#### 2.4.2. Compressive Strength Test

The compressive strength of cement paste specimens was examined on an NYL-300D mechanical test machine (Wuxi Jianyi Instrument & Machinery Co., Ltd; Wuxi, China). The specimens were compressed by the machine on an area of 40 mm × 40 mm to obtain the compressive strength with a curing age of 28 days. The temperature and loading speed for the compressive strength test were 20 °C and 500 N/s, respectively. Meanwhile, three specimens were prepared in each group. Finally, the error of data should not exceed 15.0% of the average value, and the average value was considered as the test result.

#### 2.4.3. Apparent Contact Angle Test

**Figure 2:**
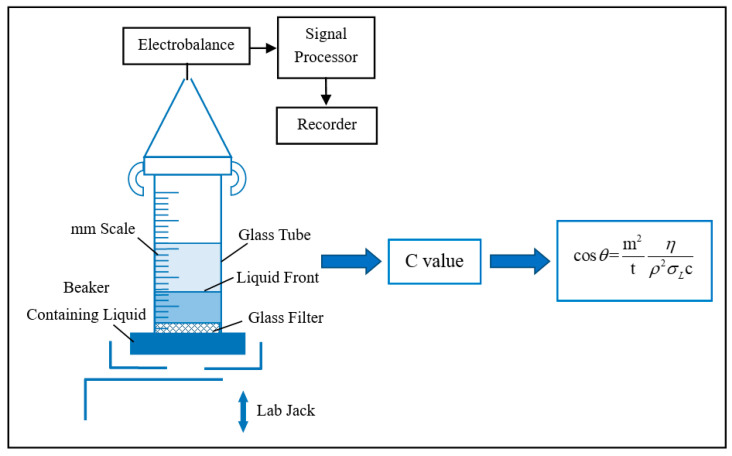
Washburn sorption method.

The modified cement powder was composed of cement and 4.0% IPEs. Then, it was dried for the apparent contact angle test. The dry modified cement powder was examined on a DACAT 21 dynamic contact angle measuring instrument. Figure 2 shows the test principle. The C value (capillary coefficient) was determined by the measurement of n-hexane, and the apparent contact angle of the cement powder was calculated by the C value (Figure 2). In addition, the instrument range for the apparent contact angle test was 0°–90°.

#### 2.4.4. Impermeability Test

The impermeability of cement mortar with a curing age of 28 d was tested according to the Standard for Test Method of Basic Properties of Construction Mortar (JGJ/T 70-2009) [20]. First, the peripheries of six specimens in each group were sealed with sealing materials. Second, sealed specimens were placed into a cement mortar permeameter for the impermeability test. Then, the water pressure was started at 0.2 MPa and then increased to 0.3 MPa after maintaining a constant pressure for 2 h. Then, the water pressure was increased by 0.1 MPa every hour. The test could be stopped, and the water pressure at that time could be recorded when three of six specimens exhibited seepage on the end face. During the impermeability test, the test should be stopped, and the peripheries of specimens should be sealed again if the water seeped out from their peripheries. Then, the resealed specimens were placed again into the cement mortar permeameter to continue the impermeability test. The impermeability pressure value of specimens was calculated by the maximum pressure of four of six specimens in each group when seepage was not observed, as shown in Equation (1).

(1) P=H−0.1

where, *P* is the permeability pressure value of cement mortar, MPa, and *H* is the maximum water pressure among three of six specimens with permeation, MPa.

#### 2.4.5. Microstructure Tests

In this work, two microstructure tests were conducted: transmission and reflection polarizing microscope (TRPM) test and environmental scanning electron microscopy (ESEM) test. For the TRPM test, IPEs were first spread uniformly on glass slides. Then, the glass slide was placed on an objective table for observation by TRPM at a temperature of 25 °C.

For the ESEM test, the effect of IPEs on the microstructure of the cement paste with a curing age of 28 d was investigated using an S-4800 cold-field ESEM. First, the specimen was cut into small particles, followed by their examination at an applied accelerating voltage of 5.0 kV and a temperature of 25 °C.

## 3. Correlation Analyses

### 3.1. Non-Linear Correlation Analysis and Linear Correlation Analysis

Non-linear correlation and linear correlation are common data analysis methods. In several practical problems, the relationship between variables is non-linear. Generally, there is some curve relationship between variables. For this relationship, a non-linear correlation analysis method for analyzing variables is utilized. Generally, a nonlinear regression model is expressed in Equation (2).

(2) $$Y=\varphi (x_{1},x_{2},\ldots ,x_{m},\beta_{1},\beta_{2},\ldots ,\beta_{r})+\varepsilon$$

For a given set of observations (*x_i_*,*y_i_*), *i* = 1,2,…,*n*, Equation (2) can be revised as Equation (3).

(3) $$y_{i}=f(x_{i},\theta )+\varepsilon_{i},i=1,2,\ldots ,n$$

where, *y_i_* is a dependent variable; non-random variable *x_i_* = (*x_i_*_1_, *x_i_*_2_, …, *x_ik_*)’ is an independent variable; *θ* = (*θ*_0_,*θ*_1_, …, *θ_p_*)’ is an unknown parameter vector; and *ε_i_* is a random error term, which satisfies the independently identical distribution; that is, Equation (4).

(4) $$\left\{ \begin{array}{l} E(\varepsilon_{i})=0,i=1,2,\ldots ,n\\ cov(\varepsilon_{i},\varepsilon_{j})=\left\{ \begin{array}{l} \sigma^{2},i=j\\ 0,i\neq j \end{array}\right.(i,j=1,2,\ldots ,n) \end{array}\right.$$

Then, Equations (5) and (6) were used to solve nonlinear least-squares estimation (minimum $\hat{\theta }$) of *θ*.

(5) $$Q(\theta )=\sum_{i=1}^{n}{\left(y_{i}-f(x_{i},\theta )\right)}^{2}$$

(6) $${\frac{\partial Q}{\partial \theta_{j}}|}_{q_{j}={\hat{q}}_{j}}^{j}=-2\sum_{i=1}^{n}\left(y_{i}-f(x_{i},\hat{\theta })\right){\frac{\partial f}{\partial \theta_{j}}|}_{\theta_{j}={\hat{\theta }}_{j}}=0,(j=0,1,2,\ldots ,p)$$

where, the regular expressions (Equation (6)) can be established by the differential method to solve *Q(θ)* (to obtain minimum $\hat{\theta }$), when the *f* function is assumed to be continuous differentiable for *θ*. Let the partial derivative of the *Q* function to *θ_j_* be 0 (Equation (6)). Equation (6) is generally solved by the Newton iterative method. In addition, $\hat{\theta }$ can be calculated by minimizing the residual sum of squares.

In this work, Origin 8.5 software was utilized to establish the non-linear fitting and linear fitting. The correlation coefficient *R*^2^ was used for non-linear correlation analysis and linear correlation analysis. *R*^2^ is a dimensionless quantity with a value between −1 and 1. When *R*^2^ < 0, the correlation between variables is negative. When *R*^2^ > 0, the correlation between variables is positive. In addition, |*R*^2^| indicates the degree of correlation between variables. Generally, when 0.8 < |*R*^2^| < 1.0, the correlation between the two variables is extremely strong. When 0.6 <|*R*^2^| < 0.8, the correlation between the two variables is strong. When 0.4 < |*R*^2^| < 0.6, the correlation between the two variables is moderate. When 0.2 < |*R*^2^| < 0.4, the correlation between the two variables is weak. When 0 < |*R*^2^| < 0.2, the correlation between the two variables is extremely weak or uncorrelated.

### 3.2. Gray Correlation Analysis

Gray correlation analysis is a multi-factor statistical method that can analyze the degree of correlation among multiple factors [21,22]. The details are as follows:

(1) The reference sequence *X*_0_*(k)* and the comparative sequence *X_i_(k)* are selected from the data obtained from various experiments, as shown in Equation (7).
  (7) $$\begin{array}{l} X_{0}=X_{0}\left(1\right),\;X_{0}\left(2\right),\;\ldots ,\;X_{0}\left(n\right)\\ X_{1}=X_{1}\left(1\right),\;X_{1}\left(2\right),\;\ldots ,\;X_{1}\left(n\right)\\ \ldots \\ X_{m}=X_{m}\left(1\right),\;X_{m}\left(2\right),\;\ldots ,\;X_{m}\left(n\right) \end{array}$$
  where, *k* = 1, 2, 3, …, *n*, *i* = 1,2,3,…,*m*.
(2) To reduce the influence of the difference between the maximum and minimum values in the sequence, the sequence needs to be normalized, as shown in Equation (8).
  (8) $$x_{i}(k)=\frac{X_{i}(k)}{\frac{1}{n}\sum_{i}=1^{n}X_{i}}(k)$$
  where, *i* = 0,1,2,3,…,*m*.
(3) The gray correlation coefficient is calculated by Equation (9).
  (9) $$x_{i}(k)=\frac{\underset{i}{min}\underset{k}{min}\left|x_{0}(k)-x_{i}(k)\right|+r\underset{i}{max}\underset{k}{max}\left|x_{0}(k)-x_{i}(k)\right|}{\left|x_{0}(k)-x_{i}(k)\right|+r\underset{i}{max}\underset{k}{max}\left|x_{0}(k)-x_{i}(k)\right|}$$
  where, $\left|x_{0}(k)-x_{i}(k)\right|=D_{i}(k)$ is called the absolute deviation between *x*_0_ and *x_i_* at point *k*; $\underset{i}{min}\underset{k}{min}\left|x_{0}(k)-x_{i}(k)\right|$ is called the tow-grade minimum difference; $\underset{i}{max}\underset{k}{max}\left|x_{0}(k)-x_{i}(k)\right|$ is called the tow-grade maximum difference; and *ρ* is the distinguishing coefficient, generally *ρ* = 0.5.
(4) The relational degree is calculated by Equation (10).
  (10) $$r_{i}=\frac{1}{n}\sum_{k=1}^{n}x_{i}(k)$$
(5) According to the principle of the gray correlation degree analysis, the comparative sequence with a high relational degree is the closest to the reference sequence, which is the most important factor that affects the reference sequence.

## 4. Results and Discussion

### 4.1. Characterizations of IPEs

#### 4.1.1. FTIR Analyses

**Figure 3:**
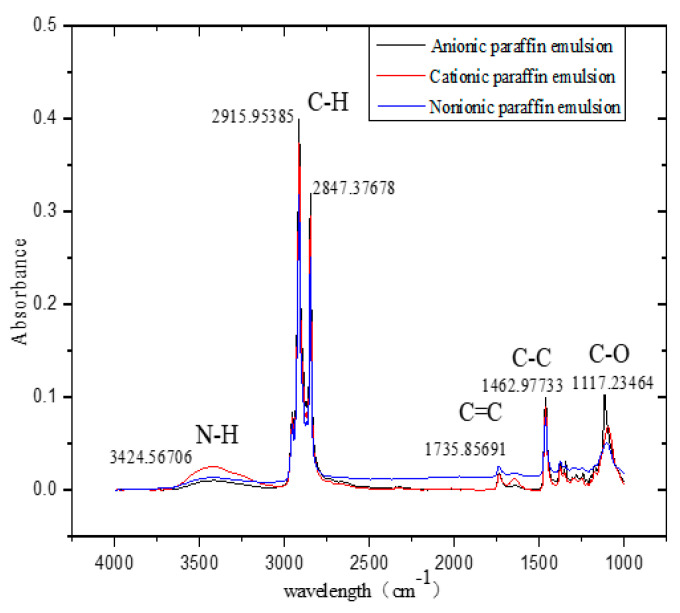
Fourier transform infrared (FTIR) spectroscopy of IPEs.

A strong peak is observed at 3000 cm^−1^ to 2750 cm^−1^, mainly corresponding to the stretching vibration of the C–H bond in IPEs (Figure 3). Peaks are observed at 1500 cm^−1^ to 1000 cm^−1^, characteristic of stretching vibrations of C–C and C–O bonds. Weak peaks corresponding to the stretching vibrations of N–H and C=C bonds are observed at 3500 cm^−1^ to 3250 cm^−1^ and 2250 cm^−1^ to 1500 cm^−1^, respectively. In addition, IPEs mainly comprise hydrophobic hydrocarbon groups, which are predominantly produced by the straight-chain hydrocarbon structure in paraffins. Meanwhile, IPEs contain a small amount of the hydrophilic amide group (–CONH–), indicating that IPEs with an excellent emulsion property can be stably distributed in the water phase. Moreover, similar peak locations are observed for anionic paraffin emulsion (APE), cationic paraffin emulsion (CPE), and non-ionic paraffin emulsion (NPE). However, the peak intensities of APE, CPE, and NPE are slightly different due to different ionic emulsifiers.

#### 4.1.2. Microstructural Analyses

**Figure 4:**
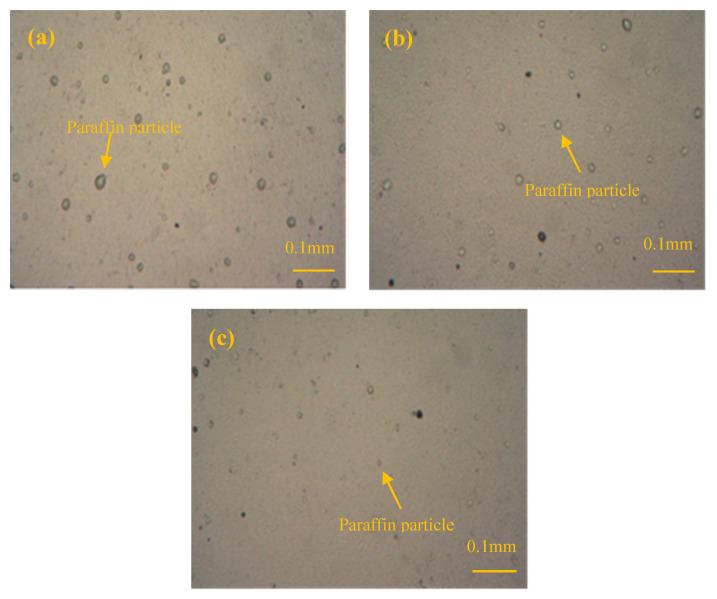
Transmission and reflection polarizing microscope (TRPM) photographs of IPEs (×100): (**a**) anionic paraffin emulsion (APE), (**b**) cationic paraffin emulsion (CPE) and (**c**) non-ionic paraffin emulsion (NPE).

The particle size of IPEs was observed by TRPM (Figure 4). Paraffin particles are small and uniformly distributed, demonstrating an excellent emulsion property; this property is conducive to the mixing of cement-based materials with IPEs. Notably, NPE exhibits the lowest particle size, which is more conducive to the distribution of paraffin particles of NPE in cement-based materials.

### 4.2. Properties of Cement-Based Materials with IPEs

#### 4.2.1. Compressive Strength

**Figure 5:**
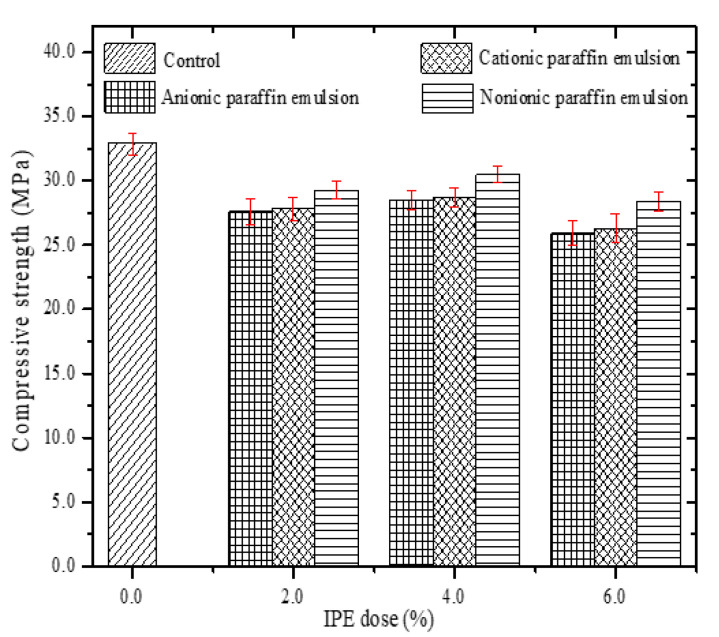
Compressive strength of cement paste with different IPE doses.

**Figure 6:**
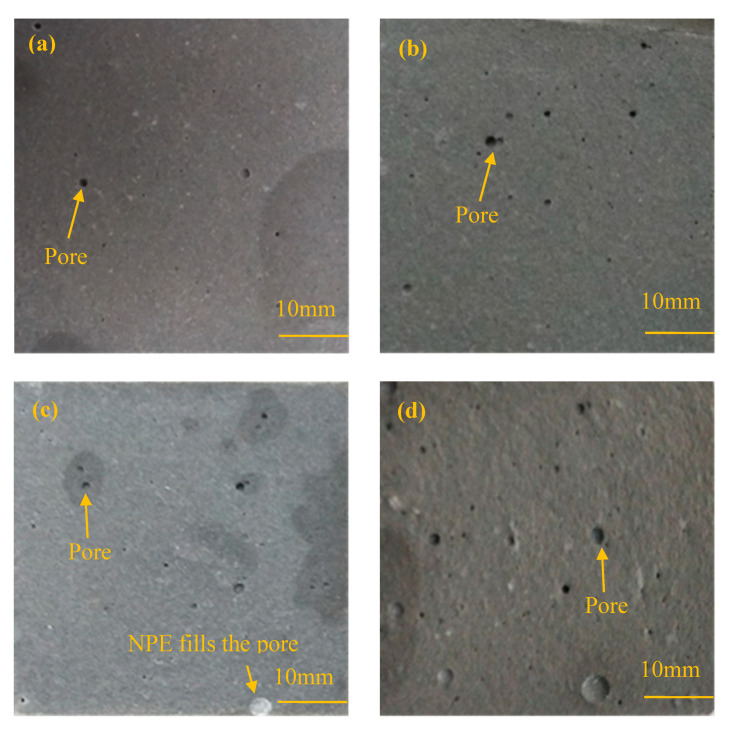
Digital photographs of cement paste with different NPE doses: (**a**) 0%, (**b**) 2.0%, (**c**) 4.0% and (**d**) 6.0%.

Figure 5 shows the change in the compressive strength of cement paste specimens with different IPE doses at a curing age of 28 days. Generally, the compressive strength of cement paste specimens with IPEs is less than that of the control group. Based on the observation in Figure 5, the compressive strength of cement paste specimens increases with the increase in the IPE dose when the IPE dose is less than 4.0% (except the control group). Second, the compressive strength of cement paste specimens decreases with the increase in the IPE dose when the IPE dose is greater than 4.0%. Figure 6 shows digital photographs of cement paste specimen sections with different doses of NPE. The number of pores decreases in the following order: Figure 6d > Figure 6b > Figure 6c > Figure 6a. Meanwhile, the pore is filled by NPE in Figure 6c.

The change in the compressive strength and number of pores in cement-based materials is related to the following facts:

(1) Paraffin particles can affect cement hydration, leading to a higher number of internal pores in the cement paste specimens with IPEs than that in the control group. Therefore, the compactness of cement paste specimens with IPEs decreases, leading to a lower compressive strength of cement paste specimens with IPEs than that of the control group.
(2) Paraffin particles mainly play a role in filling the internal pores of cement paste specimens to reduce the porosity and improve the compressive strength at an IPE dose of less than 4.0% (except the control group). Thus, the compressive strength of cement paste specimens increases with the IPE dose.
(3) Excess paraffin particles adsorbed on the cement surface can affect cement hydration when the IPE dose is greater than 4.0%. Moreover, the network connection among the cement hydrates is affected due to paraffin particles, which increases the porosity of cement paste specimens. Therefore, the compressive strength of cement paste specimens decreases with the increase in the IPE dose.

#### 4.2.2. Impermeability Results

**Figure 7:**
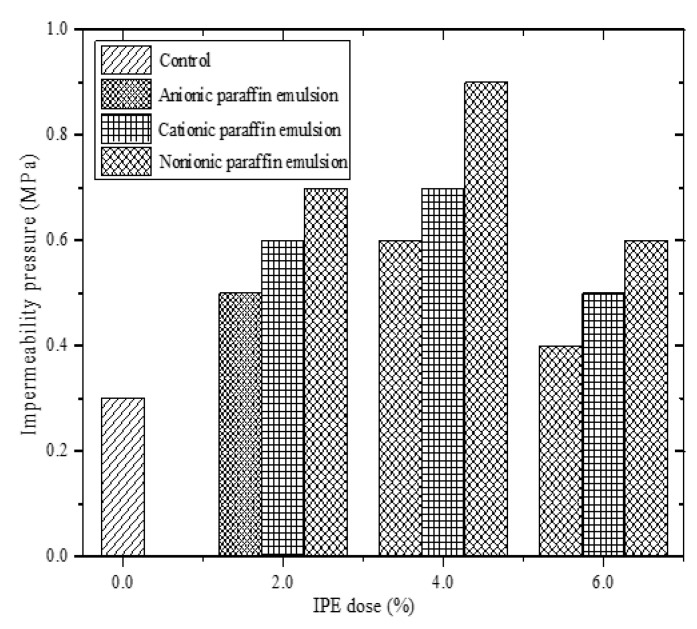
Impermeability pressure of cement mortar with different IPE doses.

**Figure 8:**
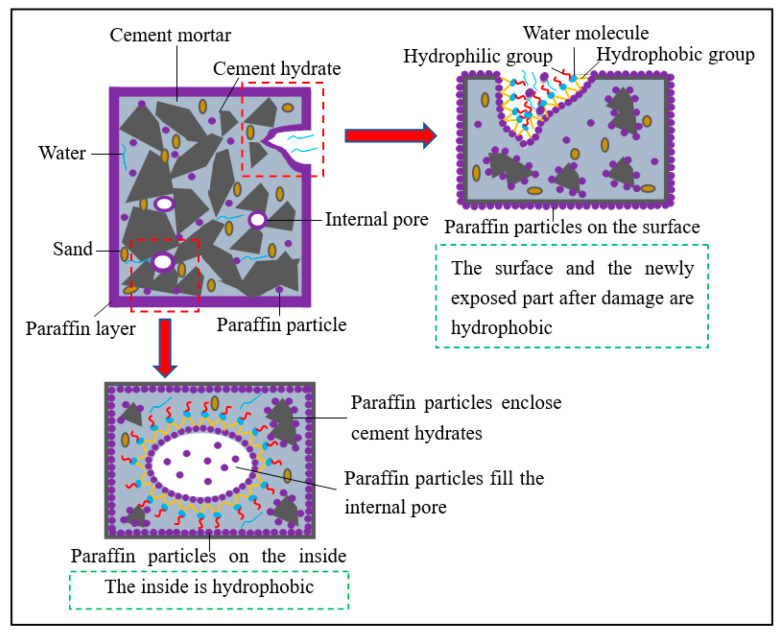
Diagram of internal and external hydrophobicity.

Figure 7 shows the change in the impermeability pressure of cement mortar specimens with different IPE doses at a curing age of 28 d. The impermeability pressure of cement mortar specimens with IPEs is higher than that of the control group, which is mainly related to the hydrophobicity of IPEs. Correspondingly, paraffin particles can fill internal pores of cement mortar specimens and enclose cement hydrates to impart hydrophobicity to the inside of cement mortar specimens; on the other hand, the IPE coating can prevent water penetration, and the newly exposed part of cement mortar specimens after damage is hydrophobic (Figure 8). In addition, the impermeability pressure clearly increases first and then decreases with the increase in the IPE dose. Hence, the IPE dose of the cement mortar is extremely high to retard the cement hydration, leading to the decrease in impermeability.

Combined with the compressive strength and impermeability tests, notably, cement mortar specimens and cement paste specimens with 4.0% NPE exhibit the highest impermeability and highest compressive strength (except the control group), respectively, which is mainly related to the non-ionic property of NPE. Therefore, NPE is not easily adsorbed on the cement surface, leading to a lower adverse effect on cement hydration than APE and CPE. Besides, hydrophilic groups of NPE mainly comprise –CONH– (oxygen-containing functional groups), leading to the strong adsorption of NPE on water molecules. Thus, NPE is conducive to the hydration of the surrounding cement. Correspondingly, the compactness of specimens with NPE is much better than that of specimens with APE and CPE, leading to a higher compressive strength and impermeability for specimens with NPE than those for specimens with APE and CPE.

#### 4.2.3. Apparent Contact Angle

**Figure 9:**
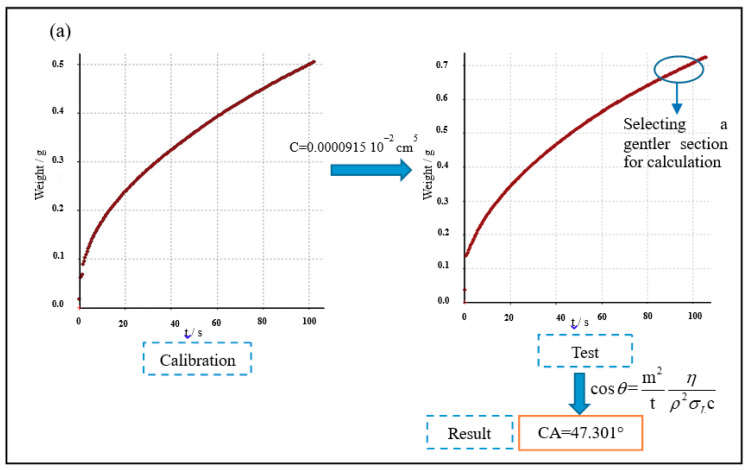

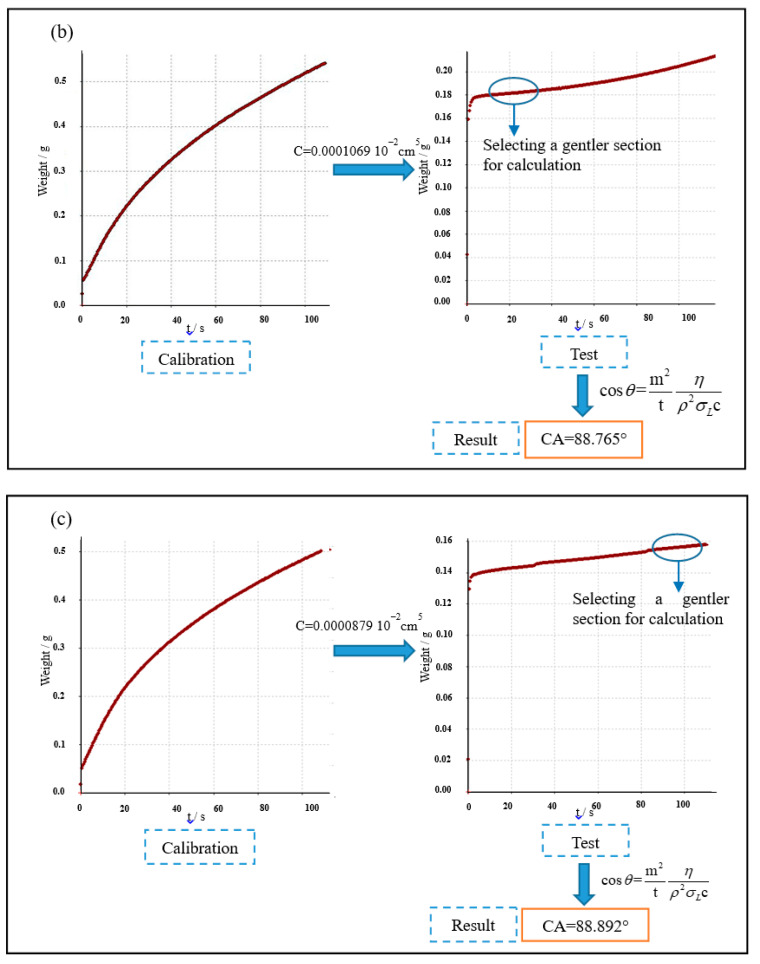

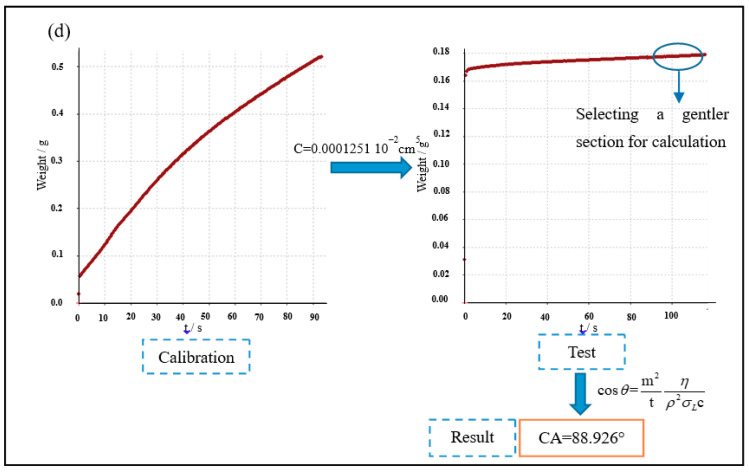
Apparent contact angle value of cement powder with 4.0% IPEs: (**a**) cement powder, (**b**) cement powder with APE, (**c**) cement powder with CPE and (**d**) cement powder with NPE.

According to the above test results, the apparent contact angle test for the cement powder with 4.0% IPEs was carried out (Figure 9). The apparent contact angle of the control group is 47.301°. The apparent contact angles of the cement powder with APE, CPE, and NPE are 88.765°, 88.892°, and 88.926°, respectively. Notably, the average apparent contact angle of the cement powder with IPEs is increased by 88.86%. Therefore, the cement powder with IPEs exhibits excellent hydrophobicity. In addition, the apparent contact angle of the cement powder with NPE is higher than that of the cement powder with APE and CPE. Thus, the hydrophobicity of the cement powder with NPE is higher than that of the cement powder with APE and CPE.

#### 4.2.4. Microstructures

**Figure 10:**
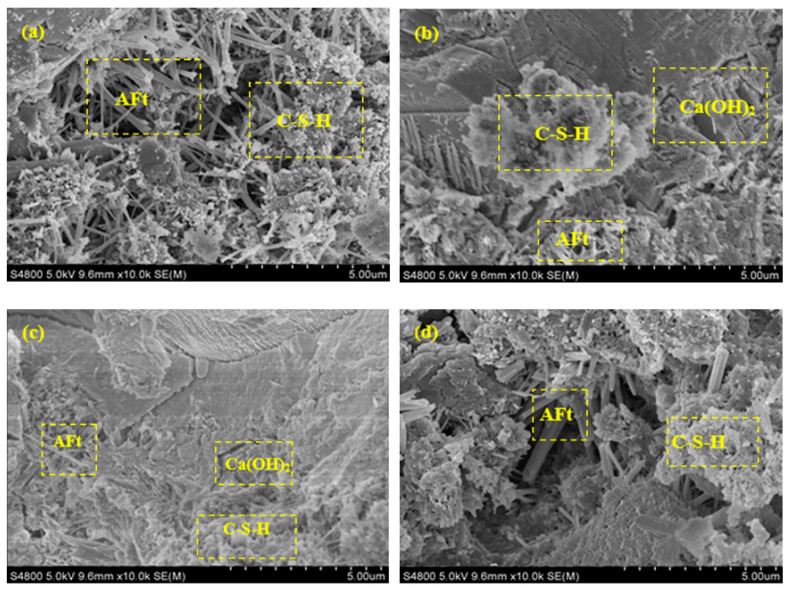
Scanning electron microscopy (SEM) photographs of cement paste with 4.0% IPEs at 28 days curing ages: (**a**) cement paste, (**b**) cement paste with APE, (**c**) cement paste with CPE and (**d**) cement paste with NPE.

Figure 10 shows the scanning electron microscopy (SEM) photographs of the cement paste with 4.0% IPEs at a curing age of 28 d. Cement hydrates are clearly observed in Figure 10. The amount of cement hydrates decreases in the following order: Figure 10a > Figure 10d > Figure 10b > Figure 10c. Furthermore, hydrates of the cement paste with NPE are similar to those of the control group.

The change in the cement hydrates of cement-based materials is related to the following facts:

(1) According to the Cassie–Baxter model [23,24], water does not easily wet the cement surface due to the hydrophobicity of IPEs, leading to the interception of gas on the cement surface to produce a gas film and hindering the complete hydration of cement. Thus, the amount of hydrates of the cement paste with IPEs is less than that of the control group, leading to lower compressive strength and compactness for the cement paste with IPEs than those for the control group.
(2) Paraffin particles in APE and CPE can be adsorbed on cement particles or hydrates more easily than those in NPE, which hinders the hydration of cement and decreases the compactness of cement hydrates. Therefore, the content of flaky calcium hydroxide in the cement paste with APE and CPE is greater than that in the cement paste with NPE; the contents of aciculate ettringite crystal and hydrated calcium silicate gel in the cement paste with APE and CPE are less than those in the cement paste with NPE, leading to lower compressive strength, impermeability, and apparent contact angle for the cement paste with APE and CPE than those for the cement paste with NPE.

#### 4.2.5. Correlation Analyses

**Figure 11:**
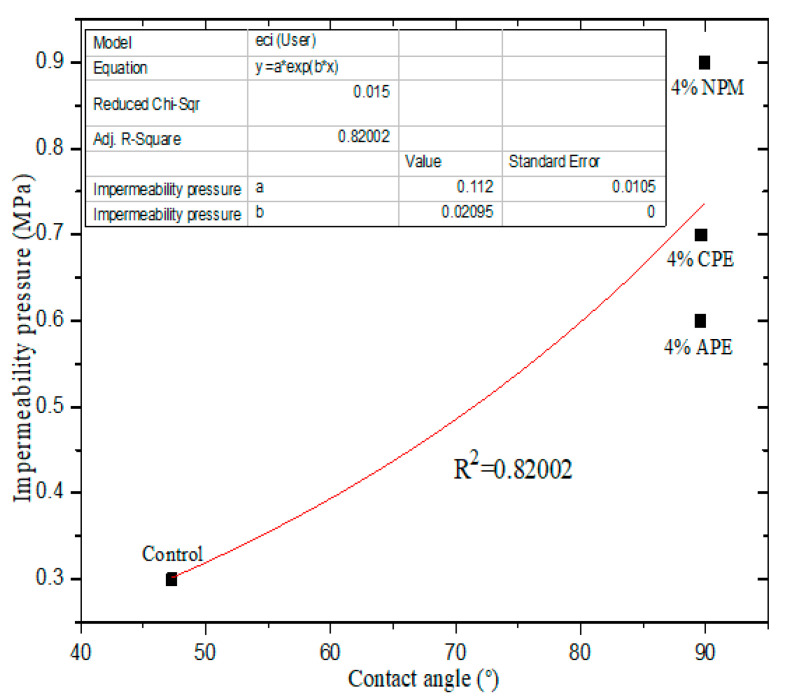
Correlation between apparent contact angle and impermeability pressure.

**Figure 12:**
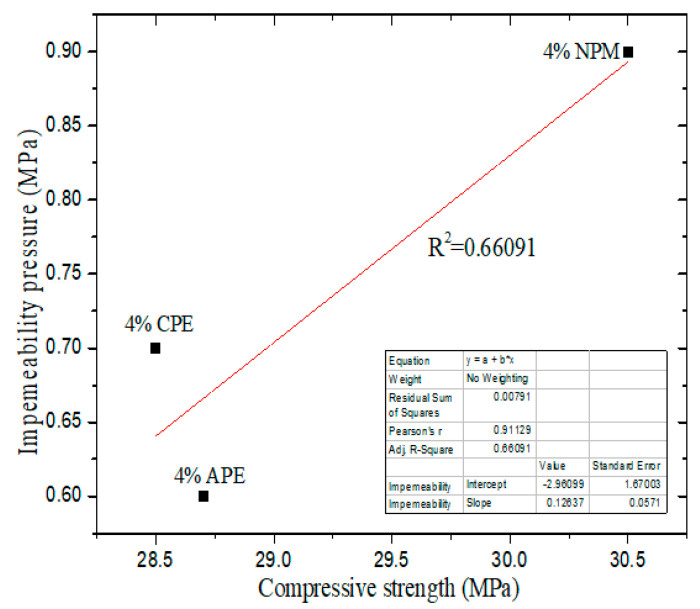
Correlation between compressive strength and impermeability pressure with IPEs.

In this work, the impermeability pressure of cement-based materials with 4% IPEs was selected as the reference sequence (X_0_), the compressive strength of cement-based materials with 4% IPEs was selected as a comparative sequence (X_1_), and the apparent contact angle of cement-based materials with 4% IPEs was selected as a comparative sequence (X_2_). Table 6, Figure 11 and Figure 12 show the results of the gray correlation analysis, non-linear correlation analysis, and linear correlation analysis, respectively.

Table 6 shows that *r*_1_ (0.77376) is greater than *r*_2_ (0.55648), which indicates that the apparent contact angle is the main factor that affects the impermeability. The apparent contact angle is positively correlated with the impermeability pressure. The higher the apparent contact angle, the higher the impermeability pressure, that is, the stronger the hydrophobicity of cement-based materials (Figure 11). In addition, the compressive strength is positively correlated with the impermeability pressure of cement-based materials with IPEs (Figure 12). However, from Figure 5 and Figure 7, the compressive strength for the control group is high, but its impermeability is not necessarily high. Therefore, the control group is not shown in Figure 12. Meanwhile, attention should be focused on the compressive strength when considering the impact of impermeability and apparent contact angle on the hydrophobicity of cement-based materials.

## 5. Conclusions and Recommendations

In this work, the effect of cement-based materials incorporated with IPEs on the hydrophobicity was examined by macroscopic and microscopic tests as well as correlation analyses. The effect of IPEs on the hydrophobicity improvement of cement-based materials was analyzed. In addition, the optimal type and the recommended dose of IPEs were suggested. The following conclusions and recommendations can be drawn:

(1) The impermeability of cement mortar specimens with IPEs reflects exceptional hydrophobicity. Notably, cement mortar with 4.0% NPE exhibits outstanding hydrophobicity.
(2) The apparent contact angle of cement powder with IPEs is significantly higher than that of the control group, indicating that IPEs exhibit excellent hydrophobicity. Notably, the apparent contact angle of cement powder with NPE is near 90°.
(3) The microstructures of cement paste with NPE are clearer than those of the cement paste with APE and CPE. Meanwhile, the microstructures of cement paste with NPE are similar to that of the control group.
(4) The apparent contact angle is the main factor that affects the impermeability. Notably, attention should be focused on the compressive strength when considering the effect of impermeability and apparent contact angle on the hydrophobicity.
(5) The compressive strength of cement-based materials with 4.0% NPE is considerably similar to that of the control group. However, NPE adversely affects the compressive strength. Therefore, we recommend that NPE can be mixed with mineral admixtures to improve the compressive strength of cement-based materials.

## 6. Further Research

Water can influence the cement hydration and concrete durability with different curing ages. Suitable water absorption can be beneficial for consolidating the concrete with positive effects in its resistance properties. However, excessive or short water absorption can result in the protection deterioration of concrete. Therefore, it is necessary to study the influence of the introduction of NPE on concrete durability in further research.

## Figures and Tables

**Table 1:** Properties of composite Portland cement.

Density/(g/cm^3^)	Average Particle Size/μm	Setting Time/min		Flexural Strength/MPa		Compressive Strength/MPa		Main Chemical Composition/%	
		Initial	Final	3d	28d	3d	28d	CaO	SiO_2_
3.150	20.786	141	233	3.8	6.2	18.9	35.8	60.1	20.8

**Table 2:** Properties of fine aggregate.

Density/(g/cm^3^)	Mud Content/(%)	Fineness Modulus
2.600	1.5	2.76

**Table 3:** Properties of fully refined paraffin.

Melting Point/(°C)	Containing Oil/(m/m)	Penetration Degree/(25 °C, 100g, 1/10mm)	Color Number	Water-Soluble Acid or Alkali	Mechanical Impurities and Moisture
52–54	0.55	15	+30	None	None

**Table 4:** Mix proportion of cement paste specimens.

IPE (%)	Cement (g)	Water (g)	IPE (g)
0.0	1350.0	472.5	0
2.0	1350.0	456.3	27.0
4.0	1350.0	440.1	54.0
6.0	1350.0	423.9	81.0

Note: the dose of IPE is calculated according to the percentage of cement mass.

**Table 5:** Mix proportion of cement mortar specimens.

IPE (%)	Cement (g)	Sand (g)	Water (g)	IPE (g)
0.0	675.0	1012.5	236.3	0
2.0	675.0	1012.5	228.2	13.5
4.0	675.0	1012.5	220.1	27.0
6.0	675.0	1012.5	212.0	40.5

Note: the dose of IPE is calculated according to the percentage of cement mass.

**Table 6:** Results of correlation.

Correlation	Properties	Impermeability Pressure
Gray correlation	Apparent contact angle	0.77376
	Compressive strength	0.55648
Non-linear correlation	Apparent contact angle	0.82002
Linear correlation	Compressive strength	0.66091
